# Apparent diffusion coefficient maps in the assessment of surgical patients with lumbar spine degeneration

**DOI:** 10.1371/journal.pone.0183697

**Published:** 2017-08-28

**Authors:** Evgenii Belykh, Andrey A. Kalinin, Arpan A. Patel, Eric J. Miller, Michael A. Bohl, Ivan A. Stepanov, Liudmila A. Bardonova, Talgat Kerimbaev, Anton O. Asantsev, Morgan B. Giers, Mark C. Preul, Vadim A. Byvaltsev

**Affiliations:** 1 Laboratory of Neurosurgery, Irkutsk Scientific Center of Surgery and Traumatology, Irkutsk, Russia; 2 Department of Neurosurgery, Irkutsk State Medical University, Irkutsk, Russia; 3 Department of Neurosurgery, Barrow Neurological Institute, St. Joseph’s Hospital and Medical Center, Phoenix, Arizona, United States of America; 4 Department of Spinal Surgery and Pathology of Peripheral Nervous System, JSC “National Scientific Center of Neurosurgery”, Astana, Kazakhstan; University of Chicago, UNITED STATES

## Abstract

**Purpose:**

To assess the utility of apparent diffusion coefficient (ADC) maps for the assessment of patients with advanced degenerative lumbar spine disease and describe characteristic features of ADC maps in various degenerative lumbar spinal conditions.

**Methods:**

T1-weighted, T2-weighted and diffusion weighted (DWI) MR images of 100 consecutive patients admitted to the spinal surgery service were assessed. ADC maps were generated from DWI images using Osyrix software. The ADC values and characteristic ADC maps were assessed in the regions of interest over the different pathological entities of the lumbar spine.

**Results:**

The study included 452 lumbar vertebral segments available for analysis of ADCs. Characteristic ADC map features were identified for protrusion, extrusion and sequester types of lumbar disk herniations, spondylolisthesis, reactive Modic endplate changes, Pfirrmann grades of IVD degeneration, and compromised spinal nerves. Compromised nerve roots had significantly higher mean ADC values than adjacent (p < 0.001), contralateral (p < 0.001) or adjacent contralateral (p < 0.001) nerve roots. Compared to the normal bone marrow, Modic I changes showed higher ADC values (p = 0.01) and Modic 2 changes showed lower ADC values (p = 0.02) respectively. ADC values correlated with the Pfirrmann grading, however differed from herniated and non-herniated disks of the matched Pfirrmann 3 and 4 grades.

**Conclusion:**

Quantitative and qualitative evaluation of ADC mapping may provide additional useful information regarding the fluid dynamics of the degenerated spine and may complement standard MRI imaging protocol for the comprehensive assessment of surgical patients with lumbar spine pathology. ADC maps were advantageous in differentiating reactive bone marrow changes, and more precise assessment of the disk degeneration state. ADC mapping of compressed nerve roots showed promise but requires further investigation on a larger cohort of patients.

## Introduction

Low back pain is an important socioeconomic and health problem of the modern society and a major cause of disability in adults of working age [1]. The most frequent causes of low back pain include various degenerative pathological conditions of the lumbar spine, for example lumbar disk herniations, spondylolisthesis, degenerative disk disease, many of which have indications for surgery when the conservative treatment fails to provide relief.

Magnetic resonance imaging (MRI) is widely used for imaging evaluation of patients with low back pain. T1 and T2-weighted MRI sequences (T1-weighted and T2-weighted) provide anatomical information regarding the soft tissues primarily; including fat and water content and are usually used for the assessment of the lumbar discovertebral complex. Evaluation of T1 and T2 sequences is focused on the structural changes in the intervertebral disk and zygapophyseal joints, reactive vertebral bone marrow changes, location and extent of the disk material displacement, degree of the stenosis, location and extent of nerve root compression. Diffusion weighted imaging (DWI) is a standard MRI sequence for the brain; however, it is not often used for imaging of the spine. A series of DWIs taken at different b values, a measure of the gradient magnetic field strengths, can be used to calculate an apparent diffusion coefficient (ADC) map, which shows the relative speed by which water can diffuse through a tissue. Nutrient diffusion limitation has been elucidated as an important pathophysiological mechanism of disk degeneration, contributing significantly to biomolecular and cellular changes that develop as part of the degenerative pathology [2, 3]. Besides its use for intervertebral disk, assessment of the tissue diffusion phenomenon may improve our understanding of other normal and pathological features of degenerating spine and bring new insight to the diagnosis and treatment of degenerative spinal disorders [2, 3]. Some specific uses of ADC maps in regards to discovertebral complex include analysis of bone mineral density of lumbar vertebrae [4], diagnosis of a lumbar vertebral chordoma [5], or response of myeloma to treatment [6].

Previous studies focused on the assessment of ADC maps in healthy IVDs [7] or age [8] and degeneration [9] related ADC changes. Such studies were performed on healthy volunteers or patients seen in outpatient settings [7]. The purpose of this study was to retrospectively assess the utility of ADC mapping in pre-operative patients with various known spinal conditions and to determine if they may provide information that is clinically relevant to the preoperative assessment of lumbar degenerative spinal disease. Additionally, we quantitatively assessed ADC maps of the patients with vertebral bone marrow changes and compromised neve roots.

## Materials and methods

### Study population

The study protocol was approved by the “Local Ethics Committee of the Irkutsk Scientific Center of Surgery and Traumatology”. All patients provided written voluntary informed consent for their participation in the study. Pre-operative lumbar spine MR imaging was used to investigate 100 consecutive patients who were operated on for various pathologies in the lumbar spine. Patient’ characteristics are presented in the Table 1. Study included all consecutive patients scheduled for the surgery on the lumbar spine. We excluded patients with the spinal pathologies at the levels other than lumbar, tumors and vascular pathology.

**Table 1:** Patients characteristics.

**Total number of patients**	100
**Sex**	
Males, n (%)	89/100 (89%)
Females, n (%)	11/100 (11%)
**Age**, years	42.6±10.4
**Total IVDs assessed on MRI, n**	494
**Total IVDs with available ADC maps, n**	452
**Prevalence of pathology among IVDs assessed**	
Disk herniation, n (%)	114/494 (23%)
Spondylolisthesis, n (%)	14/494 (3%)
Lumbar Stenosis, n (%)	17/494 (3%)
Central	13/17 (6%)
Foraminal	4/17 (4%)
**Types of IVD herniation**	
Protrusion, n (%)	67/114 (59%)
Extrusion, n (%)	34/114 (30%)
Sequester, n (%)	13/114 (11%)
**Location of IVD herniation**	
Central, n (%)	30/114 (26%)
Paramedian, n (%)	64/114 (56%)
Foraminal, n (%)	19/114 (17%)
Extraforaminal, n (%)	1/114 (1%)
**Side of IVD herniation**	
Central, n (%)	28/114 (25%)
Left, n (%)	48/114 (42%)
Right, n (%)	38/114 (33%)
**Level of IVD herniation**	
L1-2, n (%)	1/114 (1%)
L2-3, n (%)	2/114 (2%)
L3-4, n (%)	15/114 (13%)
L4-5, n (%)	44/114 (39%)
L5-S1, n (%)	52/114 (46%)

IVD, intervertebral disk; ADC, apparent diffusion coefficient.

### MRI parameters

Imaging was performed on a 1.5T Siemens Magnetom Essenza scanner (Siemens Healthineers, Erlangen, Germany). Sagittal T1-weighted imaging (WI), T2-WI, and DWIs were collected for the lumbar spine of each patient, with a consistent 30 x 30 cm field of view and 4 mm slice thickness. T1-WIs were collected at a repetition time (TR) of 552 ms, echo time (TE) of 11 ms, no averages, and a matrix size of 320 x 320. T2-WIs were collected at a TR of 3500 ms, TE of 87 ms, no averages, and a matrix size of 384 x 384. A series of DWIs were collected at 3 different b-values (b = 50, 400 and 800 s/mm^2^) using a body coil with a TR of 3000 ms, TE of 93 ms, 6 averages, and a matrix size of 156 x 192. Average scan time was 6 minutes 30 seconds.

### Image processing

ADC maps were generated from the series of DWI images using the "ADCmap" plugin (http://web.stanford.edu/~bah/software/ADCmap) in Osyrix (R) software. Sagittal ADC maps, T1-WI and T2-WI were reviewed and visual qualitative patterns on ADC maps that may be valuable for the assessment of degenerative pathology of the lumbar spine were defined. ADC maps were displayed on a full dynamic scale with the Jet color look-up table. Additionally, 65% transparent ADC maps were placed over the corresponding T2-WI for better appreciation of anatomical localization.

To quantify diffusion from the lesional and normal spinal structures, the regions of interest (ROI) were drawn over various anatomical locations on sagittal ADC maps and mean and standard deviation (SD) ADC values were calculated. Oval ROIs of 40 mm^2^ size were used to assess diffusion in IVD. Mean ADCs from the center of the nucleus pulposus quantified diffusion, and the SD characterized homogeneity of the nucleus. ADC maps were assessed in various types of lumbar disk herniations (protrusions, extrusions, sequestrations [10]), spondylolisthesis, Modic changes [11], Pfirrmann disk degeneration grades [12], central stenosis of the spinal canal, and also from the compressed spinal nerve roots. During the measurement of ADC from IVD, special attention was paid to place the ROI within the limits of the nucleus pulposus on the ADC maps. In a few cases with significantly collapsed disk ROI was flattened and reduced to minimum 30 mm^2^ in order to fit the limits of the disk space. ADC measurements on the nerve roots were performed by the raters blinded to the information about the clinically affected nerve root. Image review and measurements of ADC values were performed by 3 neurosurgeons specialized in spinal surgery with 5–15 years of experience and familiar with generation and interpreting of ADC maps. Three neurosurgeons collectively evaluated all images on the same database and agreed on the ROI locations for the ADC measurements.

### Statistical analysis

Statistical analysis was performed in Statistica (Dell Inc., Tilsa, OK, USA) software. Data presented as means and standard deviations. Nonparametric tests were used to calculate coefficients of significance p: Wilcoxon matched pairs test (W), Kruskal-Wallis ANOVA (K-W), Mann-Whitney U test (U), and Spearman rank R correlation. Differences were considered significant when p < .05.

## Results

**Fig 1:**
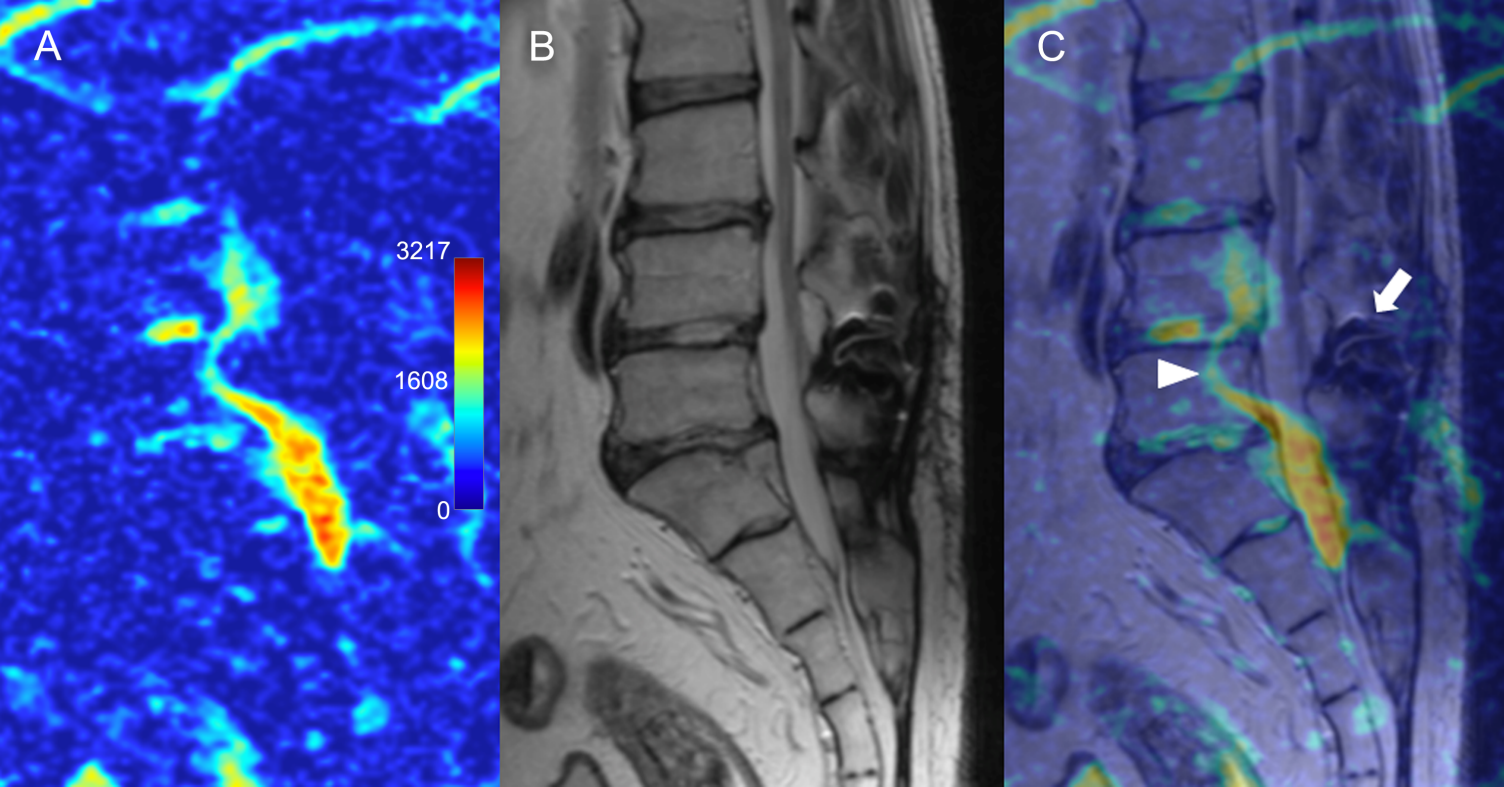
Possible artifacts on apparent diffusion coefficient maps. (A) Sagittal ADC map and (B), corresponding T2-WI and (C) colored overlay showing distortion of the signal (arrowhead) caused by previously implanted metal interspinous device (arrow).

ADC maps were generated successfully for all patients. The CSF signal had a mean ADC of 2752±333 x10^-6^ mm^2^/s. The ADC maps were calculated for the lower lumbar levels from L3 to S1 in all patients, L2-3 in 93/100 (93%) patients, L1-2 in 59/100 (59%) patients, resulting in 452 vertebral segments available for diffusion analysis. Eight segments were excluded from the analysis due to the image artifacts (bending of the DWIs image signal that changed the ADC values significantly). Metal spinal implants introduced imaging artifacts and so corresponding segments were excluded from the analysis (Fig 1).

### Types of lumbar disk herniations

**Fig 2:**
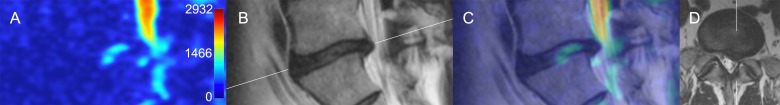
Protrusion type of disk herniation. (A) Sagittal ADC map and (B), corresponding T2-WI and (C) colored overlay bulging annulus fibrosus and contained nucleus pulposus of L5-S1 disk. (D)–axial T2-weighted image showing location of the disc herniation.

**Fig 3:**
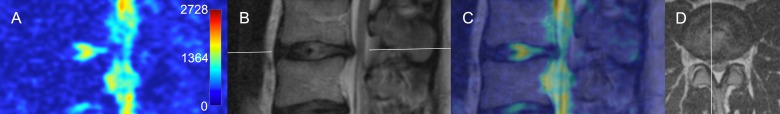
Protrusion type of disk herniation. (A) Parasagittal ADC map, corresponding T2-WI (B), colored overlay (C) and axial T2-WI (D) showing L3-4 disk bulging with a horizontal hyperintense line on a T2-WI and ADC map representing fissure in the annulus fibrosus. Also note hyperintense ADC signal from the posterior longitudinal ligament which represents enlarged venous plexus.

Disk herniations (n = 114) were classified as protrusion (n = 67), extrusion (n = 34) or sequester (n = 13) type based on the T2-WIs. ADC/T2-WI overlay images provided additional visual information about the composition of disk tissue. Sagittal ADC maps allowed for a visual differentiation of the protrusions composed of bulging annulus fibrosus (Fig 2), from the protrusions with annular clefts and protruded nucleus (Fig 3). ADC maps increased contrast and allowed for better differentiation of annular clefts and protruding nucleus when such distinction was not clear on a T2-WI.

**Fig 4:**
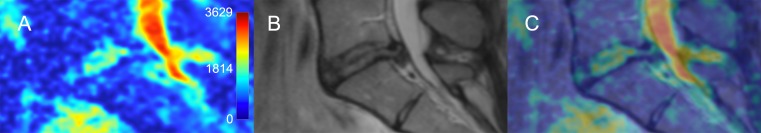
Extrusion type of disk herniation. (A) Parasagittal ADC map, (B) corresponding T2-WI, and (C) colored overlay showing L5/S1 disk with extrusion composed of hyperintense central part (extruded nucleus pulposus) and hypointense edges (ruptured annulus fibrosus).

Extruded herniations were clearly visible on both T2-WI and ADC maps. Interestingly, ADC/T2-WI composites were helpful for characterization of the extruded tissue structure and for defining the extent of nucleus pulposus extrusion under the posterior ligaments (Fig 4).

**Fig 5:**
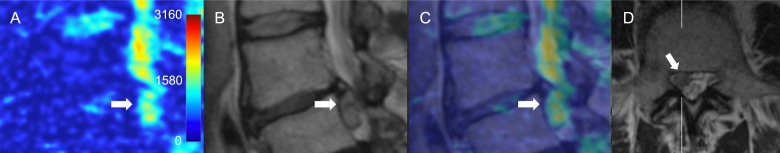
Sequester type of disk herniation. (A) Parasagittal ADC map, (B) sagittal T2-WI, (C) overlay and (D) axial T2-WI showing caudally migrated T2 hyperintence disk fragment occupying paraforaminal zone ADC, apparent diffusion coefficient; T2-WI, T2-weighted image.

Recently extruded sequesters retained mean ADC value of the nucleus pulposus (Fig 5); however, in some older sequesters the mean ADC value of the nucleus pulposus increased. For instance, in one representative example, sequester ADC value was higher than the ADC value from the adjacent non-herniated L3-4 nucleus pulposus (1665 ± 381 vs. 1449 ± 273 x1^-6^ mm^2^/s, p < 0.05). Among the all herniated disks (n = 114, all Pfirrmann grades included in analysis), there were no differences in the mean ADC values measured on the center of the disk when compared between the various LDH types (p_K-W_ = 0.13), various LDH locations (p_K-W_ = 0.74) and various LDH levels (p_K-W_ = 0.77).

Shmorl's herniations were found in 50 lumbar disks (44 single and 6 double per disk) of 28/100 (28%) patients. Single Shmorl’s nodes were found in 15 patients, two in n = 5 patient, three in n = 5 patients, four in n = 2 patients, and eight in one patient. There were no differences in mean ADC values from the disks with and without Shmorl’s nodes when all disks were assessed together (p = 0.92) and when compared in subgroups of vertebral levels (L1-2 p = 0.94, L2-3 p = 0.57, L3-4 p = 0.57, L4-5 p = 0.49, L5-6 p = 0.33) or types of LDH (protrusions p = 0.38, extrusions p = 0.51, sequester p = 0.95).

### Compromised spinal nerve roots

**Fig 6:**
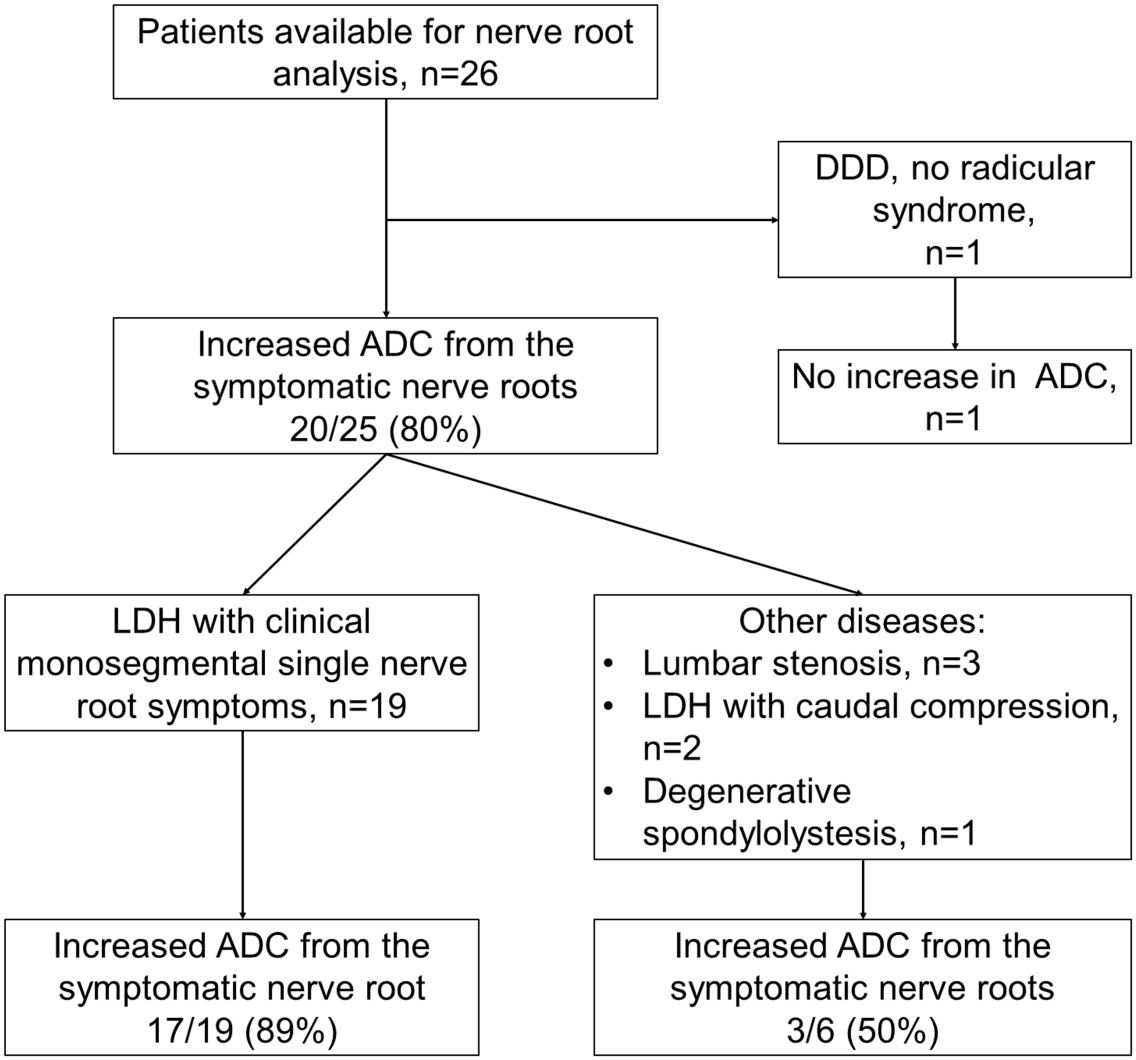
Flow chart of patients available for nerve root ADC analysis. Of the patients with monoradicular symptoms, majority had increased ADC values from the compromised nerve root comparing to the neighboring adjacent and contralateral nerve roots.

**Fig 7:**
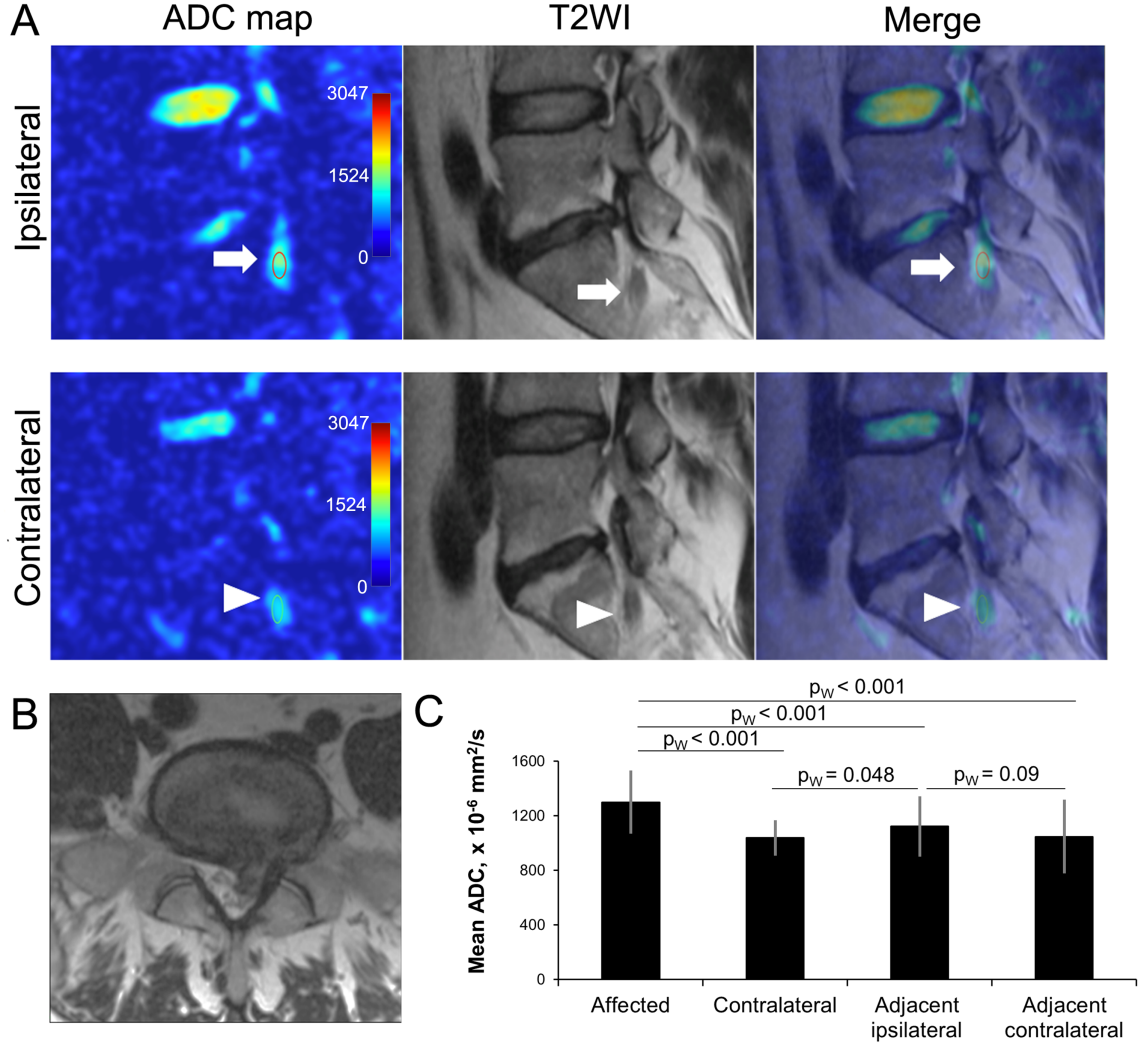
Assessment of nerve root ADC. (A) ADC maps, T2-WI and merged images of left (ipsilateral) and right (contralateral) parasaggital lumbar scans of 38 y.o. male patient with right sided L5-S1 disk herniation. Regions of interest (15 mm^2^) were selected over the ipsilateral (arrows) contralateral (arrowheads) S1 nerve roots. (B) Axial T2-WI showing disk herniation. (C) Comparison of ADC values from the affected, contralateral, adjacent ipsilateral (S1 or L5), and adjacent contralateral nerve roots. Data calculated from 19 patients with symptomatic disk herniations. *—p < .01. ADC, apparent diffusion coefficient; T2-WI, T2-weighted image.

Direct comparison of ipsilateral and contralateral ADC maps showing spinal nerve roots and ganglion inside and just outside of the spinal foramina was possible in 26 patients (Fig 6). Of those patients, 19 had monosegmental radiculopathy due to lumbar disk herniation. In this subgroup of patients ipsilateral mean ADC values from the compressed spinal nerve (1300±221 x10^-6^ mm^2^/s) were significantly higher than from the adjacent contralateral (1038±118 x10^-6^ mm^2^/s, p_W_ < 0.001), adjacent ipsilateral (1122±212 x10^-6^ mm^2^/s, p_W_ < 0.001), or adjacent contralateral (1048±259 x10^-6^ mm^2^/s, p_W_ < 0.001) nerve roots (Fig 7).

### Reactive vertebral bone marrow changes

Modic changes were found in 60/494 (12%) studied vertebral segments of 41/100 (41%) patients. Modic type 1 changes were present in 9/100 (9%) patients (in 9/494 (1.8%) studied lumbar segments). Modic type 2 changes were present more frequently, in 37/100 (37%) patients or in 51/494 (10.3%) of all studied lumbar segments.

**Fig 8:**
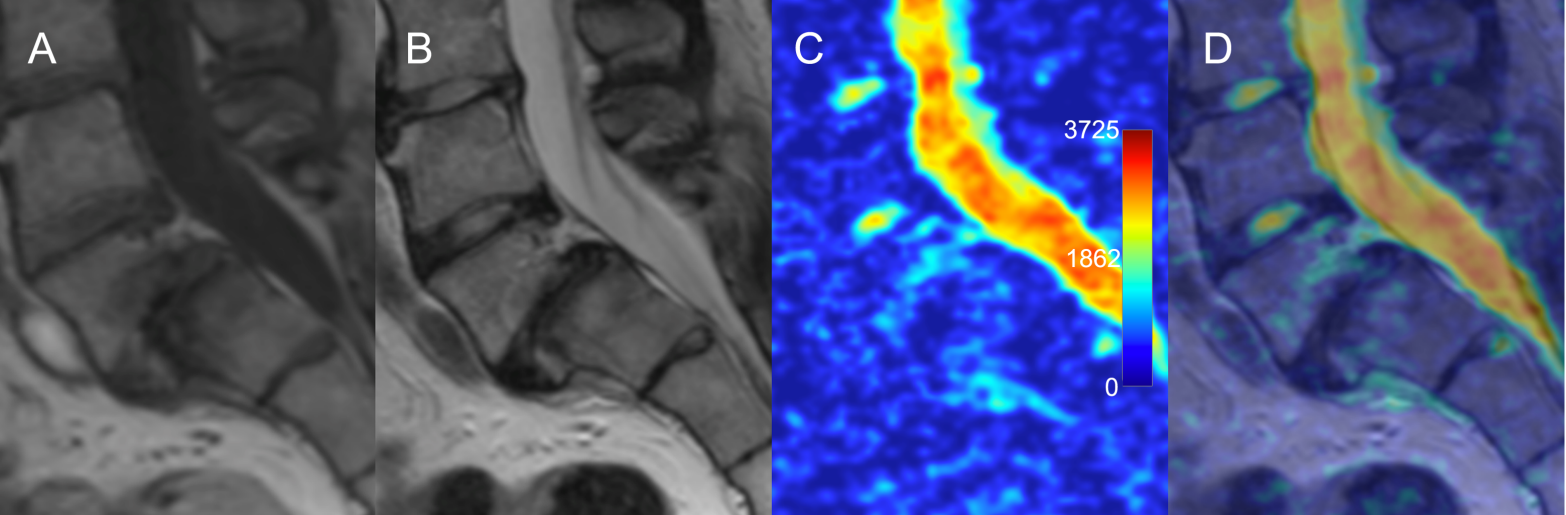
Modic type 1 changes in the setting of degenerative spondylolisthesis. (A) T1-WI shows hypointense signal; (B) T2-WI shows hyperintense signal; (C) ADC map shows hyperintense signal; (D) overlay of ADC map over T2 image depicts anatomical location of the regions with increased diffusivity corresponding to the margin of Modic 1 changes. ADC, apparent diffusion coefficient; T2-WI, T2-weighted image.

Modic type 1 changes were evident on the ADC maps (Fig 8). Mean ADC values from the Modic type 1 changes associated with degenerative spondylolisthesis were higher than from the vertebral bone marrow. Modic type 1 changes were associated with significantly higher ADC values compared to a similar ROI from the adjacent healthy vertebral bone (498 ± 139 vs. 314 ± 86 x 10^−6^ mm^2^/s, respectively, p_U_ = 0.01) and when compared to Modic type 2 changes (223 ± 110 x10^-6^ mm^2^/s, p_U_ < .001) (Table 2).

**Table 2:** Modic changes, associated ADC values and Pfirrmann grades.

	Modic 1 (n = 9)	Modic 2 (n = 51)	p_U_ value
ADC value at the corresponding bone marrow area, x 10^−6^ mm^2^/s	498±139	223±110	< 0.001
ADC value from the nearest disk, x10^-6^ mm^2^/s	1088±457	1091±397	0.82
Pfirrmann grades from the nearest disk			0.17
Grade 3, n	1	14	
Grade 4, n	4	26	
Grade 5, n	4	11	

ADC, apparent diffusion coefficient.

**Fig 9:**
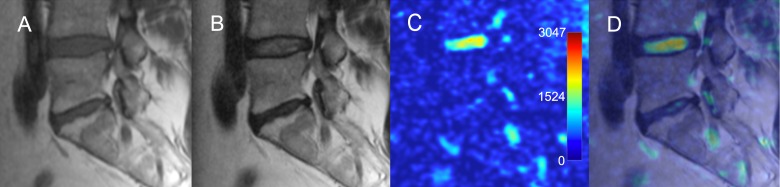
Modic type 2 changes of the vertebral bone marrow adjacent to a degenerated intervertebral disk. (A) T1-WI and (B) T2-WI show hyperintense signal. (C) ADC map and (D) overlay of ADC map over T2-WI show decreased signal intensity over the degenerated disk and over the area of reactive bone marrow changes. ADC, apparent diffusion coefficient; T2-WI, T2-weighted image.

Modic type 2 changes representing bone marrow replacement by a fat were visually similar and had significantly lower ADC values compared to the intact vertebral bone on ADC maps (p_U_ = 0.002) (Fig 9). All segments with type 2 changes showed low ADC values from both the disk area and the bone marrow. We did not observe significant changes that could be interpreted as Modic type 3 representative of subchondral bone sclerosis in the assessed patients.

### Disk degeneration

**Fig 10:**
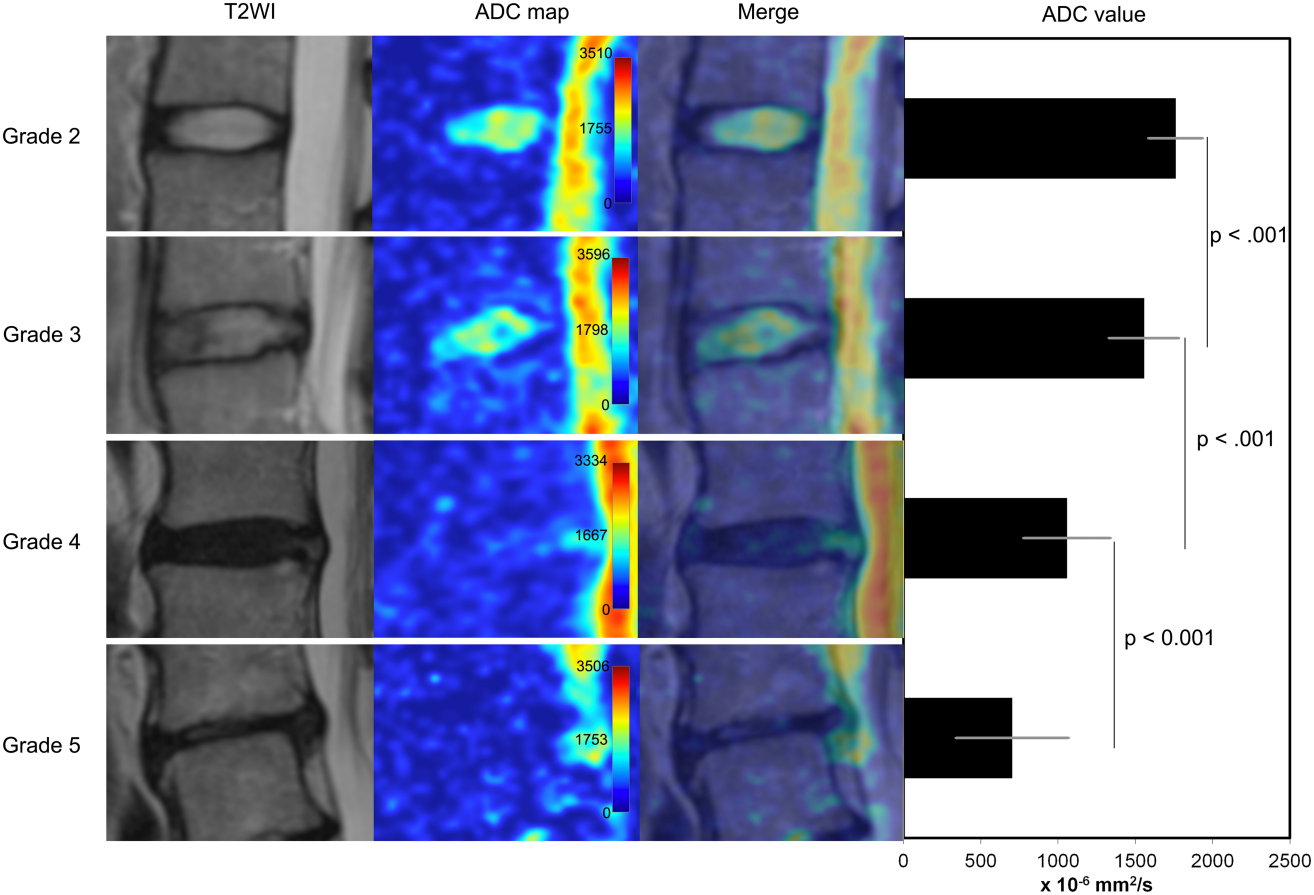
Pfirrmann disk degeneration grades and ADC maps. Appearance of various grades of disk degeneration on the ADC maps is shown on the left panel. Diagram on the right shows comparison of mean ADC values in different Pfirrmann grades (all disks included in analysis, n = 452). ADC, apparent diffusion coefficient; T2-WI, T2 weighted image.

**Fig 11:**
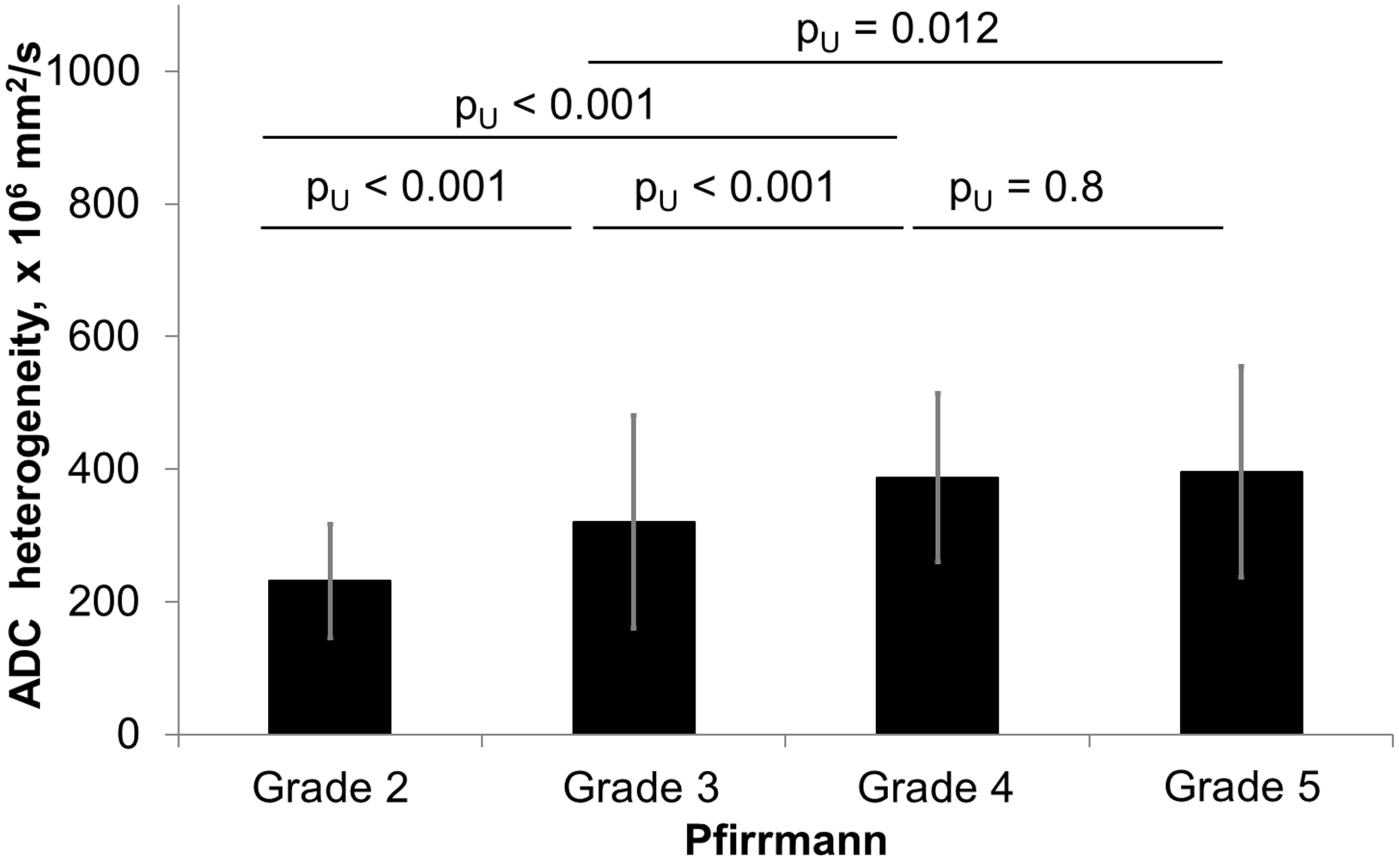
Comparison of ADC heterogeneity in various Pfirrmann degeneration grades of all intervertebral disks. Diagram showing ADC heterogeneity in intervertebral disks of Pfirrmann grades 2 through 5. 452 disks included in analysis. Significant differences were observed among all grades. ADC, apparent diffusion coefficient.

**Fig 12:**
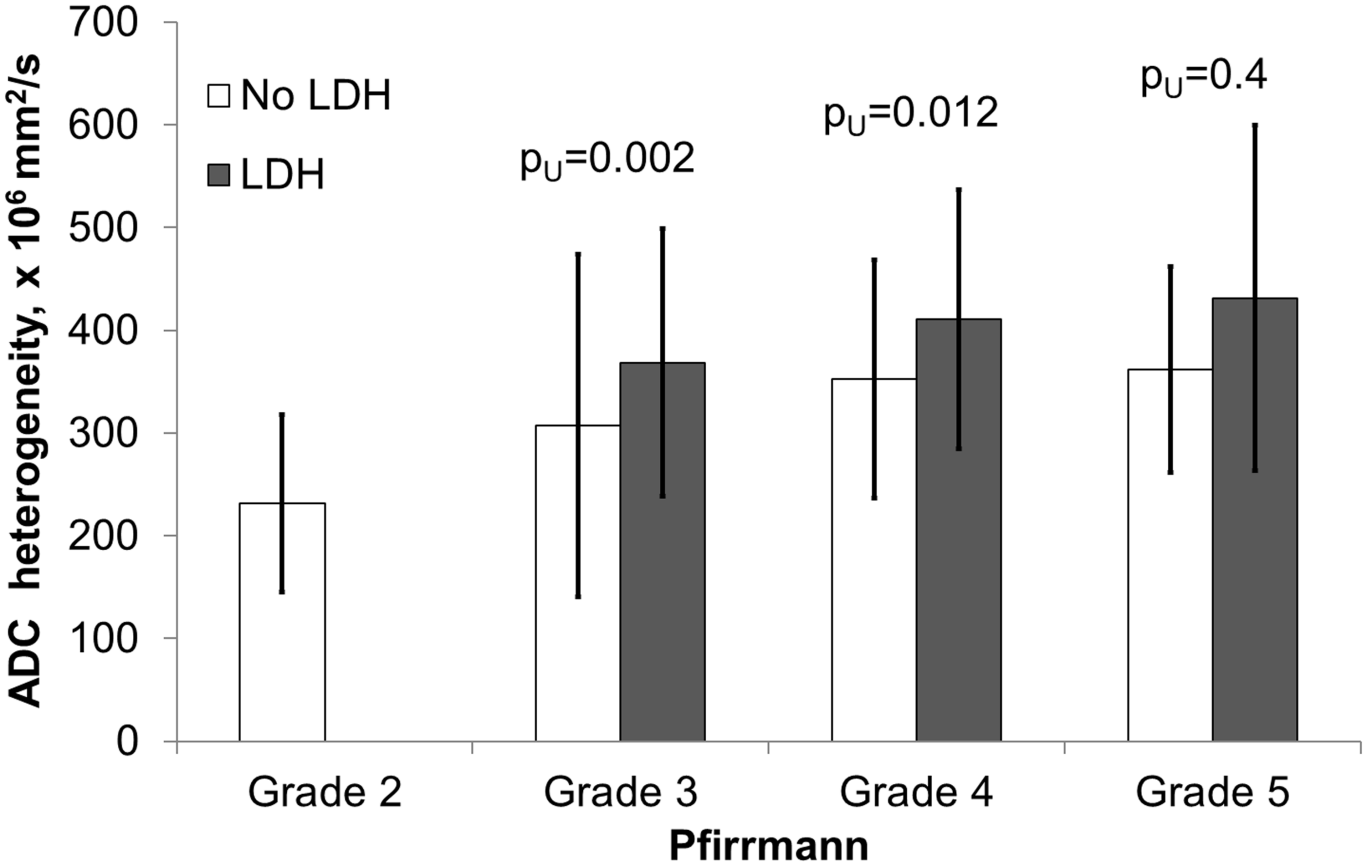
Comparison of ADC heterogeneity from herniated and non-herniated disks in various Pfirrmann grades. Diagram of ADC heterogeneity of herniated and non-herniated intervertebral disks stratified by the Pfirrmann grades. Significant differences were observed in the Pfirrmann 3 and 4 groups. Totally 114/452 (25%) disks were included in the analysis as herniated (grade 3, n = 42; grade 4, n = 57 and grade 5, n = 15), 316/452 (70%) as non-herniated (grade 2, n = 114; grade 3, n = 150; grade 4, n = 48; grade 5, n = 4), and 22/452(5%) disks were excluded from the analysis due to spondylolisthesis or lumbar stenosis without herniation. ADC, apparent diffusion coefficient, LDH, lumbar disk herniation.

**Fig 13:**
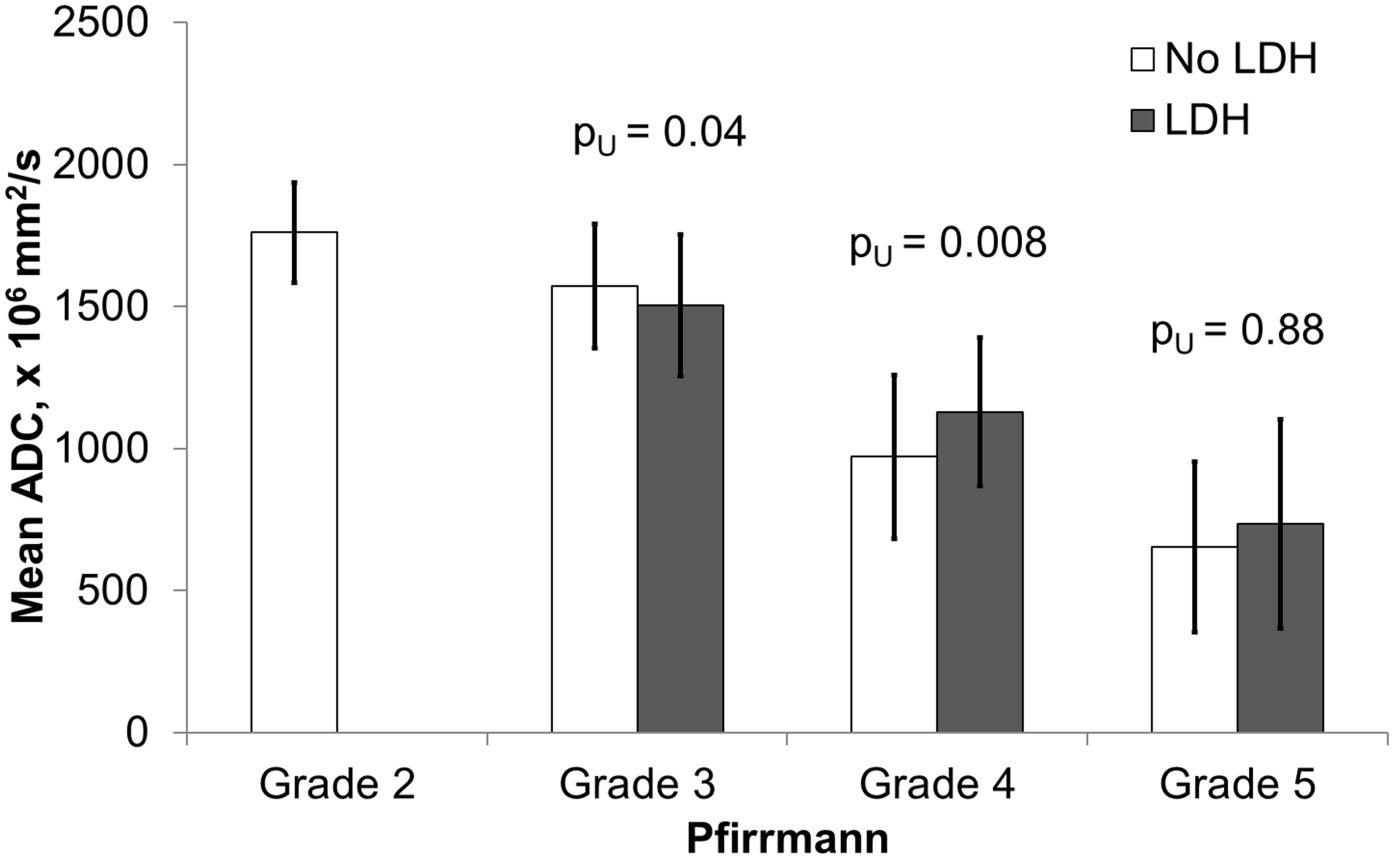
Assessment of ADC values from herniated and non-herniated disks in various Pfirrmann degeneration grades. Diagram of mean ADC values in herniated and non-herniated intervertebral disks stratified y the Pfirrmann grades. Significant differences were observed in the Pfirrmann grades 3 and 4. Totally 114/452 (25%) disks were included in the analysis as herniated (grade 3, n = 42; grade 4, n = 57 and grade 5, n = 15), 316/452 (70%) as non-herniated (grade 2, n = 114; grade 3, n = 150; grade 4, n = 48; grade 5, n = 4), and 22/452(5%) disks were excluded from the analysis due to spondylolisthesis or lumbar stenosis without herniation. ADC, apparent diffusion coefficient, LDH, lumbar disk herniation.

Quantitative assessment showed that the mean ADC values have a significant correlation with the Pfirrmann degeneration grades (Fig 10, S1 Table). Visual grading of highly degenerated disks on ADC maps was not straightforward due to the inhomogeneity of the disk. Disks with Pfirrmann grade 4 and 5 were significantly less homogeneous than grade 2 and 3 (Fig 11). Among the grade 4 disks the mean ADC values were significantly higher in herniated compared to non-herniated disks (p_U_ < 0.01), and opposite for the grade 3 disks: herniated disks had significantly lower mean ADC values than non-herniated (p_U_ = 0.04) (Fig 12). Herniated disks of grades 3 and 4 showed significantly more ADC heterogeneity than non-herniated disks of the same grades (p_U_ < 0.01 and p_U_ = 0.01 respectively) (Fig 13).

### Correlation analysis

Mean nucleus ADC values inversely correlated with spinal level, Pfirrmann grade, age, and presence of disk herniation. Nucleus ADC heterogeneity showed opposite correlations of slightly lower power with the same variables (Table 3).

**Table 3:** Correlation analysis (R Spearman coefficients).

	Mean Nucleus ADC	Nucleus ADC heterogeneity	Level	Pfirrmann grade	Age	Disk herniation (yes / no)	Shmorl’s node (yes / no)	Modic changes
Mean Nucleus ADC	1.00	-0.40*	-0.24*	-0.77*	-0.42*	-0.34*	0.00	-0.07
Nucleus ADC heterogeneity	-0.40*	1.00	0.32*	0.44*	0.21*	0.34*	0.00	-0.01
Level	-0.24*	0.32*	1.00	0.41*	0.00	0.48*	-0.12*	-0.18
Pfirrmann grade	-0.77*	0.44*	0.41*	1.00	0.34*	0.45*	0.00	-0.14
Age	-0.42*	0.21*	0.00	0.34*	1.00	-0.03	0.05	0.17
Disk herniation	-0.34*	0.34*	0.48*	0.45*	-0.03	1.00	-0.07	0.16
Shmorl’s node	0.00	0.00	-0.12*	0.00	0.05	-0.07	1.00	0.03
Modic changes	-0.07	-0.01	-0.18	-0.14	0.17	0.16	0.03	1.00

ADC, apparent diffusion coefficient, NP, nucleus pulposus;

*—p < 0.05

## Discussion

In this study we retrospectively investigated ADC maps both qualitatively and quantitatively in a selective cohort of 100 patients who underwent surgery for the treatment of degenerative lumbar spine pathology. To our knowledge, this is a first study that focuses on assessment of ADC maps of the degenerative pathology of the lumbar spine. On a large patient sample this study described characteristics of ADC images for different types of disk herniations, nerve root compression, reactive endplate changes and disk degeneration grades.

### Qualitative features of ADC maps

ADC maps overlaid onto T2-WIs provided additional contrast and information about the diffusivity of different types and locations of disk herniations. There were no overall significant differences in mean ADC values from the nucleus in different types and locations of herniations; however, this study did not investigate local changes in ADC values of different parts of the disk. Additionally, we found association between increase in diffusion heterogeneity and disk tissue degeneration. Future studies focused on the assessment of local distributions of ADCs across the various parts of the disks and adjacent structures are required to validate our qualitative visual observations in different pathological conditions of the IVD.

#### Nerve roots

**Fig 14:**
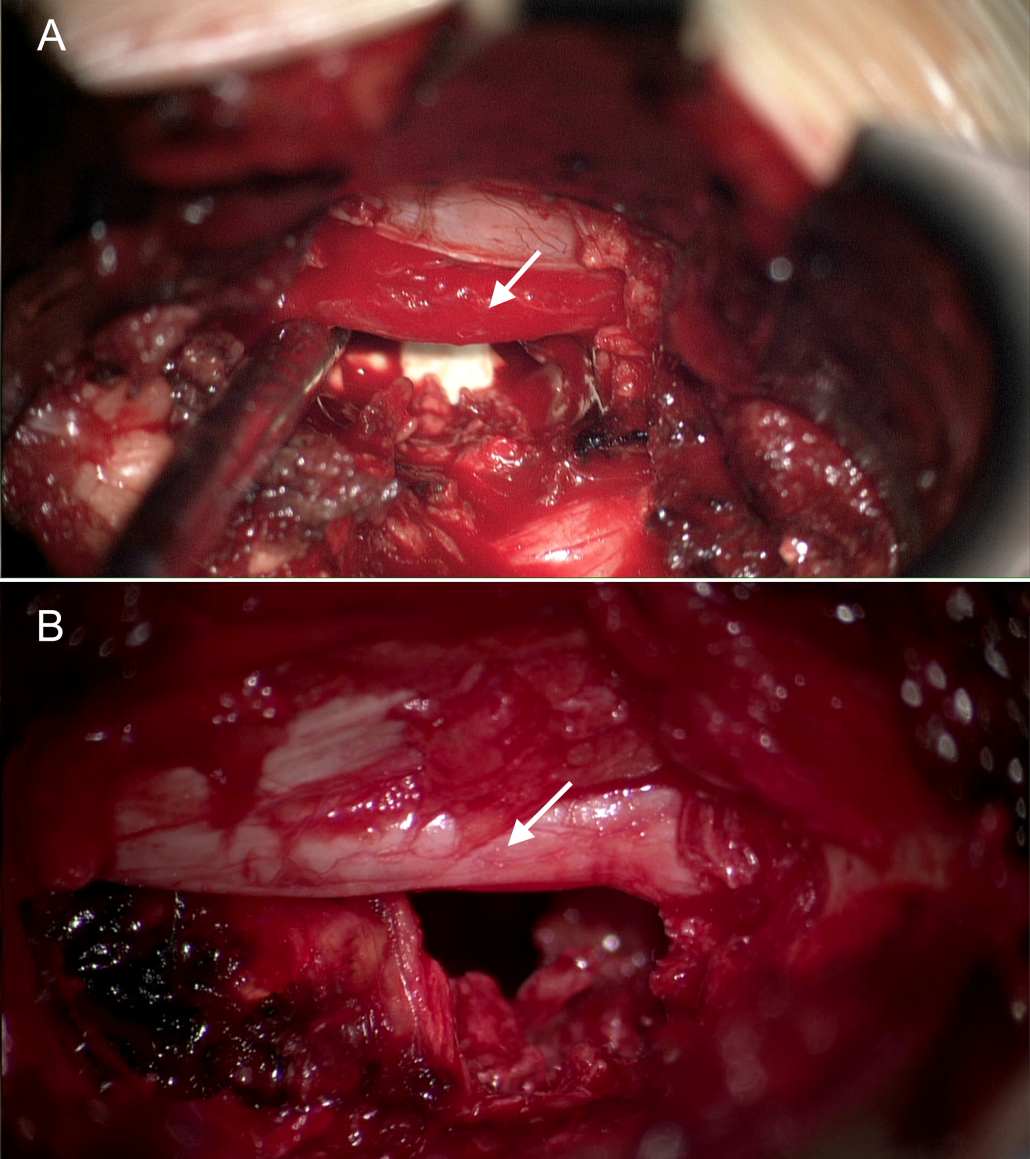
Intraoperative pictures of spinal nerve roots with various degree of inflammation. (A) 28 y.o. male with right sided L4-5 disk herniation. Representative intraoperative image after removal of the herniation shows red inflamed L5 nerve root (arrow). Right L5 nerve root ADC value is 1199 ± 113 x10^-6^ mm^2^/s; (B) 52 y.o. male M. with left sided herniated disk at L5-S1 level. Intraoperative picture after removal of the herniation shows slightly edematous S1 nerve root (arrow). Left S1 nerve root ADC value is 1030 ± 192 x1^-6^ mm^2^/s. ADC, apparent diffusion coefficient; LDH, lumbar disk herniation.

DWIs and ADC maps of spinal nerve roots, ganglions, and nerves with generation of tractography [13, 14] were shown to add value for the preoperative assessment of spinal nerve root lesions [15, 16]. However, ADC values from the dorsal root ganglion in symptomatic patients were found to be inconsistent, but correlate with recovery from pain and numbness [17]. Furthermore, not all symptomatic patients have increased ADC values in the regions of compromised nerve roots and ganglions. Interestingly, our study showed significant differences in ADC values from the symptomatic nerve roots compared to the nerve roots of the contralateral and adjacent segments, although visually ADC maps were not strikingly different. In patients with clinical signs of monosegmental radiculopathy, ADC values from the compressed nerve root were significantly higher than any neighboring nerve root (ipsilateral adjacent, contralateral on the same level, or contralateral on the adjacent level) in 17/19 (89%) patients. Only in 2 patients the adjacent ipsilateral nerve root had higher ADC values than the affected nerve root. If compare nerve roots on symptomatic level only, ipsilateral nerve root ADCs (1300±221 x 10^−6^ mm^2^/s) were on average 25% higher than contralateral (1038±118 x 10^−6^ mm^2^/s) in all 19/19 patients, p_W_ < 0.001. Furthermore, ADC values from the ipsilateral adjacent nerve roots were significantly higher compared to the contralateral nerve roots on the same level (p_W_ < 0.048), and nearly significantly higher compared to contralateral nerve roots on the adjacent level (p_W_ = 0.09). Such findings are in accordance with the frequent clinically observed ipsilateral symptoms in the LDH patients [18, 19]. Only rarely, does contralateral radiculopathy occur with LDH [20]. Additionally, we have reviewed intraoperative videos of selected cases with monosegmental radiculopathy and found that ADC values may be increased in compressed nerve roots without the typically assumed visual appearance of inflammation during surgery (Fig 14). Our findings confirm previously reported preliminary findings with a comparable number of patients [21, 22]. However, we believe that further larger study is warranted to determine the specificity and sensitivity of ADC maps for the assessment of radicular symptoms and nerve root compression. Sagittal ADC maps were used in this study for the nerve root assessment, while previous studies used axial ADC maps [15, 16]. Axial ADC maps may be easier to identify nerve roots, but weather the quantitative ADC measurements from the axial and sagittal scans are different remains unknown. At this time, our data form 26 patients suggest that increased ADC values alone would have a high positive predictive value, but low sensitivity for the diagnosis of the symptomatic spinal nerve root compression. Additionally, low visual contrast between the compressed and healthy nerves and surrounding tissues is still a limiting factor for the use of ADC maps.

#### Modic changes

This study showed that Modic type 1 changes are associated with increased ADC values, which corresponds to an increased diffusion in this region compared to the vertebral endplates without Modic changes or to the Modic type 2 changes. This finding reflects the inflammatory, edematous [23], or infectious [24] nature of the pathological substrate in Modic 1 changes. The probable “claw sign” [25] was found in all segments with the Modic type 1 changes, favoring degeneration over infection. In 5/51 (10%) segments with Modic 2 changes we have found high mean ADC values similar to what was seen in Modic type 1 changes. These emphasized the complexity of the underlying mechanisms of reactive vertebral body bone marrow changes, which do not always represent fat degeneration [26]. Modic changes were predominantly found in grade 4 and 5 degenerated disks with lower ADC values.

#### Pfirrmann grades

This study has also revealed potential limitations of the Pfirrmann grading system for the assessment of degeneration in herniated disks. The mean nucleus ADC values and the ADC heterogeneity differed significantly between the herniated and non-herniated disks in the matched Pfirrmann grades. Also, transition in ADC values from grade 3 to grade 4 is quite abrupt. We suggest that Pfirrmann grades 3 and 4 may cover an overly broad range of states of disk degeneration. Some herniated disks were assigned Pfirrmann grade 4 because of a dark inhomogeneous signal on the T2-WI. However, ADC values were significantly higher than expected for this Pfirrmann grade 4 because when the nucleus pulposus dislocates, the center of the disk contains less hydrated nuclear tissue and more annular and transition zone tissue. Indeed, the original grading scale developed by Boos and Pfirrmann [12] was described without specific attention to herniations, lysthesis, endplate changes, or discitis. Whether the freshly herniated disks cause temporary increase in local diffusion and increased ADC values remains unconfirmed. Injury to the avascular annulus fibrosis would likely not result in edema to the same extent as other more vascularized tissues. Another explanation for increased ADC values in the degenerated disk is a presence of cracks filed with an exudate [7]. Proper grading of disk degeneration is important for early diagnostics, comparison across clinical studies, and estimation of regenerative potential. Therefore, more adequate grading systems should be considered [27–29]. ADC maps may detect changes not visible on T2-WI [8] and therefore may be used as an adjunct for more precise evaluation of the disk degeneration.

#### Advantages and disadvantages of ADC maps

Overall, the benefits of ADC maps for the assessment of patients with surgical pathology of the lumbar spine include several aspects. First, the imaging of the spinal nerves may provide additional information on their precise anatomical location, possible entrapment, and potential functional prognosis. Second, ADC maps allow for more precise estimation of IVD degeneration. Third, ADC/T2-WI composites provide increased image contrast and additional functional information for the assessment of the normal and pathological anatomy of the lumbar spine. Finally, vertebral bone marrow and endplate infectious, degenerative and fatty changes could be differentiated more easily with ADC maps.

The drawbacks of diffusion imaging include the lack of the anatomical image, artifacts on the edges of the scan area, and possible patient movement during scanning. Another limitation is the additional time required for DWI sequence acquisition and ADC map processing. Automated calculation and overlay of ADC maps on the corresponding anatomical images, such as T2-WI, will make this modality more useful in the clinical settings.

#### Limitations

There are several limitations in this study. The study population represents a selected subgroup of spine surgery patients with lumbar disk herniations, spondylolisthesis and symptomatic lumbar disk degeneration. Therefore, generalization of the findings to a larger population of patients without such pathology should be done with caution. The number of patients with lysthesis was low, but we assume that the qualitative ADC map will be similar for other cases, although this should be confirmed in a larger cohort. The imaging was performed on a 1.5 T MRI with defined TE, TR, and b values. Direct comparison of the ADC values obtained with other imaging parameters may differ and should be done with caution. Calculating interobserver variability was not within the scope of this study. We deemed for this study it was better to have a discussion amongst the expert surgeons with regard to ROI placement, grading, and interpretation of herniation.

## Conclusions

This study revealed benefits and limitations of qualitative and quantitative ADC map evaluation of pre-operative lumbar spine patients using a contemporary 1.5 T MRI. ADC maps overlaid on T2-WIs provide functional information about tissue diffusion, which may be useful for assessment of the morphology of various degenerative conditions in the lumbar spine. ADC maps were advantageous for differentiating reactive vertebral bone marrow changes and for more precise assessment of the disk degeneration state. ADC mapping showed promise for detection and assessment of compressed nerve roots but this aspect requires further investigation on a larger cohort of patients.

## Supporting information

S1 Table Pfirrmann disk degeneration grades and associated ADC values from intervertebral disks. (DOCX)
