# Supplementary material for: Apparent diffusion coefficient maps in the assessment of surgical patients with lumbar spine degeneration
Source: PLoS One. 2017 Aug 28;12(8):e0183697. doi: 10.1371/journal.pone.0183697 (PMC5573303; doi:10.1371/journal.pone.0183697)
Supplement: S1 Table — (DOCX) [file pone.0183697.s001.docx]

**S1 Table. Pfirrmann disk degeneration grades and associated ADC values from intervertebral disks.**

| **Pfirrmann grade** | **Number of disks**  **(%)** | **ADC value,**  **Mean ± SD, x 1^-6^ mm^2^/s** | **ADC heterogeneity,**  **Mean ± SD, x 1^-6^ mm^2^/s** |
| --- | --- | --- | --- |
| 2 | 115/452 (25%) | 1760 ± 175 | 232 ± 86 |
| 3 | 197/452 (44%) | 1555 ± 225 | 321 ± 160 |
| 4 | 115/452 (25%) | 1056 ± 289 | 387 ± 127 |
| 5 | 25/452 (6%) | 703 ± 363 | 396 ± 159 |
| Total | 452 (100%) |  |  |

ADC, apparent diffusion coefficient.
